# Predictors and interdependence of family support in a random sample of long‐term young breast cancer survivors and their biological relatives

**DOI:** 10.1002/cam4.1766

**Published:** 2018-09-05

**Authors:** Maria C. Katapodi, Katrina R. Ellis, Franziska Schmidt, Christos Nikolaidis, Laurel L. Northouse

**Affiliations:** ^1^ Nursing Science Faculty of Medicine University of Basel Basel Switzerland; ^2^ School of Nursing University of Michigan Ann Arbor Michigan; ^3^ School of Social Work University of Michigan Ann Arbor Michigan; ^4^ Institute of Higher Education and Research in Healthcare ‐ IUFRS University Hospital Vaudois ‐ CHUV University of Lausanne ‐ UNIL Lausanne Switzerland

**Keywords:** Actor‐Partner Interdependence Model, dyads, principal component analysis, random sample, triads, young breast cancer survivors

## Abstract

**Objective:**

Women diagnosed with breast cancer younger than 45 years (young breast cancer survivors—YBCS) and their biological relatives face significant stressors. Although family support is an important coping resource, little is known about YBCS’ and relatives’ support and whether it is interdependent. The study described family support in YBCS and their biological relatives; identified demographic, clinical, and psychosocial predictors of support; and determined the interdependence of support in YBCS‐relatives family units.

**Methods:**

Data were collected from a random sample of YBCS and their first‐ or second‐degree female relatives. Actor‐partner interdependence models (APIM) explored predictors and interdependence of YBCS’ and relatives’ family support in dyads (YBCS and relative) and triads (YBCS and two relatives).

**Results:**

Among n = 310 YBCS and n = 431 first‐ or second‐degree relatives, family support was higher in triads compared to dyads. APIMs identified *actor effects* in dyads, and *actor* and *partner effects* in triads. Across all family units, YBCS’ higher self‐efficacy was associated with higher YBCS support (*actor effect*) and relative support (*partner effect*); YBCS’ prior diagnosis of depression was associated with lower YBCS and relative support (*actor* and *partner effect*); cost‐related lack of access to care was associated with lower support among YBCS (*actor effect*) and relatives (*actor* and *partner effect*).

**Conclusions:**

Family support was interdependent and was affected by self‐efficacy, depression, and access to care. Interventions should include YBCS and relatives, enhance self‐efficacy and access to care.

## 1 BACKGROUND

Breast cancer is the most prevalent female cancer worldwide, with 1.38 million new cases annually.^1^ About 25% of all breast cancer cases are diagnosed in women under 50 years old, constituting a growing clinical population of younger women with breast cancer.^2^ Early onset breast cancer presents several challenges, including tumors that are more aggressive, higher recurrence rates, and increased mortality, and is associated with genetic predisposition. First‐ and second‐degree relatives of young breast cancer survivors (YBCS) have a 2.3 and 1.5 increased relative risk for breast cancer, respectively.^3^

YBCS often report poorer outcomes compared to their older counterparts due to different stressors and social roles.^4^, ^5^ YBCS caring for young children may face additional difficulties communicating concerns and may feel responsible for transmitting an increased cancer risk to their offspring.^4^, ^5^ Caring for children and older parents, combined with the challenges of the disease, can cause additional distress, anxiety, depression, fear of recurrence, and difficulties returning to work.^4^, ^6^, ^7^ Loss of income due to inability to work can lead to additional financial stressors and lack of access to care.^5^ Thus, YBCS may need significantly more support to overcome these challenges compared to older breast cancer patients.^8^

Although biological female relatives have an elevated risk for breast cancer, they may not always cope with this risk and manage it effectively. Young women with a strong family history may have heightened perceptions of breast cancer risk, chronic depression, anxiety, and increased breast cancer worry.^9^, ^10^, ^11^ Family members are an important source of information about risk factors, genetics, and available screening and risk‐reducing strategies, especially for women from medically underserved communities.^12^ Multiple family members are likely to be involved, directly or indirectly, in appraisals regarding the magnitude of the health threat and the availability of coping resources. However, family members may perceive different levels of vulnerability and stigmatization associated with hereditary breast cancer, experience different levels of distress, and disagree about the extent of family involvement needed to reduce these stressors.^13^, ^14^, ^15^ Input from different family members affects support they are willing to give and receive to each other.

There is a need to promote long‐term coping in YBCS and relatives and to mitigate the burden of early onset breast cancer.^4^, ^5^ Family support is essential to successful coping of breast cancer patients^16^ and can decrease cancer‐related distress.^17^ Individuals involved in reciprocal relationships usually influence each other's thoughts, emotions, and coping behaviors.^18^ Yet, little is known about a possible interdependence of family support in YBCS and biological relatives, who also face an increased breast cancer risk due to heredity. The study addressed this gap in the literature. Specific aims were to describe family support in YBCS and their relatives; identify demographic, clinical, and psychosocial characteristics as predictors of family support; and determine the interdependence of support in YBCS‐relatives family units.

### 1.1 Theoretical framework

**Figure 1:**
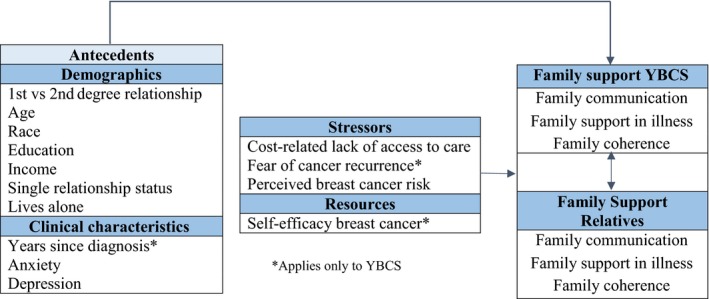
Theoretical framework

The study was guided by the integration of the theory of stress and coping^19^ with the theory of family systems in genetic illness^20^ applied to families with hereditary breast cancer risk.^21^ Stress occurs when primary appraisals of a health problem threaten one's well‐being.^19^ Primary appraisals include YBCS’ and relatives’ assessment of stressors associated with early onset breast cancer, for example, cost of health care. Primary appraisals may interfere with the ability to withstand stress because they can exacerbate YBCS’ depression and fear of cancer recurrence, and increase relatives’ perceived breast cancer risk.^22^, ^23^ Initial appraisals are followed by appraisals about the availability of personal (eg, self‐efficacy for managing breast cancer^23^) and social coping resources (eg, family support) that can help manage the health threat. Family support is the primary outcome of the study in both YBCS and their relatives (Figure 1).

## 2 METHODS

### 2.1 Design, setting, and sample

The study used baseline data obtained with a self‐administered survey for an efficacy trial designed to increase surveillance and use of cancer genetic services in YBCS and their first‐ and second‐degree relatives (ClinicalTrial.gov ID:NCT01612338).^24^ All Institutional Review Boards involved in the identification, recruitment, and consent of participants approved the study protocol. Methodological and recruitment details have been reported.^24^, ^25^ A random sample of 3000 YBCS was selected from the Michigan Cancer Surveillance Program (MCSP). Age criteria for YBCS vary among studies from 40 to 50 years old and under; we were conservative in our sample selection and we chose a cutoff of 45 years or younger at the time of diagnosis. The sample was stratified by race (1500 Black vs 1500 White/Other YBCS) to ensure an adequate representation of Black YBCS. The “Other” category includes about 7% of Michigan YBCS not recorded in the registry as Black or White (eg, Arab American). Due to their small numbers, YBCS of other racial/ethnic backgrounds could not form a separate stratum. YBCS were eligible to participate if they were diagnosed with invasive breast cancer or ductal carcinoma in situ (DCIS); were younger than 45 years old at the time of diagnosis and younger than 65 years old at the time of the study; and were willing to invite one or two relatives in the study. Relatives had to be female and in first‐ or second‐degree biological relationship with the YBCS. They had to be younger than 65 years old and cancer‐free at the time of the study.

Prior to contacting the YBCS, the director of the MCSP inquired with the reporting facility and physician of record whether there was any reason that the YBCS could not participate in the study. If MCSP did not receive a negative response within 30 days, an invitation letter explaining the study, a consent form, a self‐administered baseline survey, and a stamped return envelope were mailed to YBCS. Eligible YBCS received up to three mailed invitations to participate in the study. In order to have family units with comparable size, the study invited up to two relatives per YBCS. There were 58 YBCS carrying a mutation associated with hereditary breast cancer who were excluded from this paper; their relatives were not invited in the efficacy trial since the focus was to increase use of genetic services among untested families.

### 2.2 Instruments

The study outcome was family support in YBCS and relatives. According to the theoretical framework, predictors included stressors for YBCS (lack of access to care due to cost, anxiety, depression, and fear of cancer recurrence) and for relatives (perceived breast cancer risk), resources for YBCS (self‐efficacy dealing with breast cancer), and demographic characteristics for both YBCS and relatives.

#### 2.2.1 Family support

*Family support* was conceptualized as open communication, support in times of illness, and coherence, and was measured with three well‐established scales. All items were rated on a seven‐point Likert scale, ranging from one “Strongly Disagree” to seven “Strongly Agree.” Family communication was assessed with the Lewis Mutuality and Interpersonal Sensitivity Scale (MIS),^26^ validated with breast cancer survivors and their family members.^27^, ^28^ MIS includes 15 items (eg, “The people in my family change the topic when I discuss my concerns”); internal consistency in this study was 0.94. Family support in times of illness was assessed with the Family Support in Illness scale, originally developed for women pursuing breast cancer screening.^29^ The scale includes 10 items (eg, “In our family, when I have a health problem, there is someone helping me get the care that I need”); internal consistency in this study was 0.91. Family coherence is the ability of the family to cope with adverse events and was assessed with the Family Hardiness Index (FHI),^30^ validated with cancer and noncancer patients.^31^, ^32^ FHI includes 20 items (eg, “In our family we have a sense of being strong even when we face big problems”); internal consistency was 0.90.

A family support index was created from these three scales. Principal component analysis (PCA) examined the correlations of items (n = 45). The Kaiser‐Meyer‐Olkin measure of sampling adequacy and Bartlett's test of sphericity indicated that PCA was possible. PCA identified a primary component of family support. Pearson correlation coefficients in the component matrices ranged between 0.40 and 0.80. Four items did not correlate adequately with the principal component and were not used. An overall family support index was created by calculating a mean score from the three scales as the dependent variable.

#### 2.2.2 Stressors

*Cost‐Related Lack of Access to Care* was assessed with one item asking YBCS and relatives “Has there been a time within the past 12 months that you needed to see a doctor or have a medical test but you could not because of high out‐of‐pocket cost?” yes/no; yes indicates cost‐related lack of access to care.

*Anxiety and Depression* were assessed with two items asking YBCS and relatives “Have you ever been told by a healthcare provider that you have anxiety?” yes/no and “Have you ever been told by a health care provider that you have depression?” yes/no. These variables were assessed because they interfere with support and communication,^33^, ^34^ and better family functioning mitigates depressive symptoms among cancer patients.^35^

*Fear of Cancer Recurrence* (YBCS only) was assessed with four items from the Concerns About Recurrence Scale (CARS) (eg, “How much time do you spend thinking about your breast cancer coming back”) using a seven‐point Likert scale from one “Not at all” to seven “All the time”.^36^ Internal consistency was 0.91.

*Perceived Breast Cancer Risk* was assessed with one item asking YBCS and relatives to rate their chances of (another) getting breast cancer on a 10‐point Likert scale with verbal anchors “Definitely will not” to “Definitely will”.^37^

#### 2.2.3 Resources

Self‐efficacy managing breast cancer (YBCS only) was assessed with 14 items (eg, “Since my breast cancer diagnosis, I am able to do the things that are important for me”) scored on a seven‐point Likert scale, ranging from one “Strongly Disagree” to seven “Strongly Agree”.^38^ Internal consistency was 0.95.

*Demographic and Clinical Characteristics* such as age, education, income, living alone, relative being first vs. second degree, years since diagnosis, number of cancer diagnoses were assessed in YBCS and relatives with items from the Behavioral Risk Factors Surveillance System Survey^39^ and items developed by the team.^40^

### 2.3 Statistical analyses

Analyses were performed with SPSS^®^ version 22.0^41^ and MPlus version 7.0.^42^ Sample characteristics, stressors, and resources were described with means, standard deviations (SD), frequencies (n), or percentages (%), depending on scaling and data distribution. A *P* value ˂0.05 was considered statistically significant in all analyses. Demographics and clinical characteristics were included in all models as covariates.

Data from dyads and triads often violate the fundamental assumption of many data analyses methods that data are collected from independent subjects. YBCS and relatives have an existing interpersonal relationship; thus, correlations between YBCS’ and relatives’ data need to be taken into account. The Actor‐Partner Interdependence Model (APIM) has been used to study complex dynamics in families and close relationships.^43^ It assesses interdependence and bidirectional effects within interpersonal relationships.^44^ Observation interdependence necessitates examining the dyad (ie, the pair) as a single unit of analyses, rather than two units (ie, as single individuals). Interdependence means that observations from two or more individuals are linked. Knowledge of one's characteristics (*actor*) can provide information about the other person's (*partner*) attitudes, etc. Assessing bidirectionality involves examining each person's influence on the other person's outcomes.

**Figure 2:**
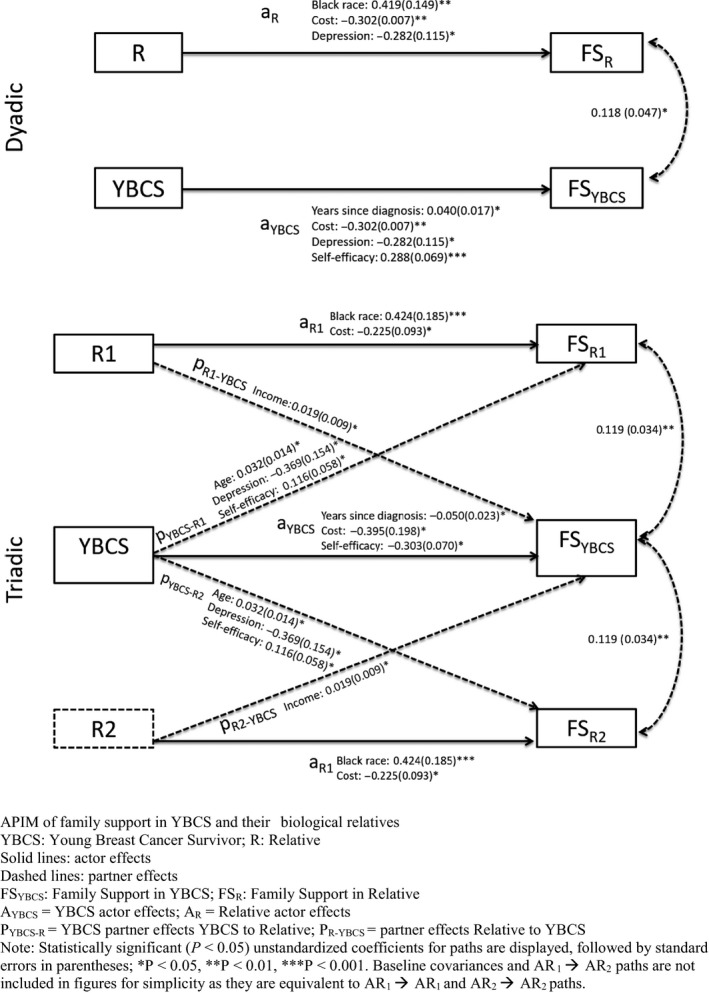
Results of actor‐partner interdependence models (APIM) of family support

We identified three types of family units for this study: dyads consisting of one YBCS and one relative; triads consisting of one YBCS and two relatives; and YBCS with no eligible relatives or whose relatives did not accept participation. The latter group was excluded from APIM analyses and this paper. APIM examined predictors of family support in dyads and triads. A dyadic model captured the interdependence of family support between YBCS and one relative (Figure 2A), and a triadic model among YBCS and two relatives (Figure 2B). *Actor effects* are observed when characteristics of one person (eg, their own resources) are significant predictors of their own outcome (ie, family support), regardless of whether this is an YBCS or a relative. *Partner effects* refer to cross‐dyadic or cross‐triadic associations and are observed when characteristics of one person in the family influence family support reported by another member.

We examined evidence of nonindependence in the outcome variable (family support index) by assessing the level of intraclass correlations.^18^, ^43^ Intraclass correlations in dyads and triads were statistically significant (0.31, 95% CI 0.17‐0.43, ANOVA *F* = 1.88, *P* < 0.001; and 0.37, 95% CI 0.23‐0.50, ANOVA *F* = 2.16, *P* < 0.001, respectively), suggesting interdependence of support in family units and that analyses should follow a dyadic and triadic format. Data were restructured in dyads including one YBCS and one relative and triads including one YBCS and two relatives. Mixed predictor variables (variables that exhibit both between‐ and within‐dyad/triad variability) were used to estimate the interdependence effects of family support.^18^, ^43^, ^44^ Full information on maximum likelihood estimation (FIML) was used in path analyses. FIML produces unbiased estimates based on all available information from variables included in an analysis, when data are missing at random or missing completely at random.

A fundamental tenet of dyadic analyses is determining distinguishability, meaning, whether or not there is a way to treat each individual as different.^18^ Distinguishable dyads are, for example, heterosexual couples; indistinguishable dyads are, for example, same‐sex roommates. We could distinguish members of dyads (YBCS and relatives), but we were unable to distinguish the two female relatives included in triads. The two relatives in triads were treated as indistinguishable, because there were no specific criteria that could designate one relative as a primary participant and the other relative as a secondary participant. Consequently, we used a novel approach for triadic analyses based on analyses methods of indistinguishable dyads. In indistinguishable dyads, data from the two individuals are pooled in order to produce parameter estimates.^18^, ^45^, ^46^ Following this approach, in the triadic APIM we pooled the estimates from the two relatives into one estimate by imposing equality constraints. Pooling the estimates from two indistinguishable relatives allowed us to estimate the effects from the two relatives as a single “relative” effect.

Indicators of adequate model fit in triadic APIM were a comparative fit index (CFI) above 0.90, a nonsignificant chi‐square statistic or a ratio of chi‐square statistic to degrees of freedom (*df*) less than two, a root mean squared error of approximation (RMSEA) value of 0.08 or less, and a standardized root mean square residual (SRMR) value of 0.08 or less.^47^, ^48^ We tested both an unconstrained dyadic model (fully saturated) and a constrained dyadic model (with actor = actor and partner = partner) constraints.^49^ A significant difference between the unconstrained and constrained model suggests that the actor effects and partner effects were equal for YBCS and relatives.

## 3 RESULTS

### 3.1 Sample characteristics

From the 3000 identified YBCS, n = 883 accepted participation (response rate 33.2% after excluding deceased, missing current address etc.). From these 883 YBCS, n = 573 participated in the study alone, either because they had no eligible relatives or because their relatives did not accept study participation.^25^ To address APIM analyses, in this paper we include only those YBCS, n = 310, who participated in the study with one or two relatives.

The 310 YBCS and their 431 blood female relatives constituted 189 dyads (one YBCS and one relative) and 121 triads (one YBCS and two relatives). Most family units (n = 249) included only first‐degree relatives (n = 164 dyads; n = 85 triads); 32 family units included only second‐degree relatives (n = 25 dyads; n = 7 triads); 29 triads were mixed, with both first‐ and second‐degree relatives. YBCS were on average 51 years old at the time of the study and 11 years postdiagnosis; approximately one in five (19.7%) had more than one cancer diagnoses. Most YBCS were White/Other, had an annual household income less than $80 000, had some college‐level education or above, and were married. Relatives were on average 43 years old; most were White/Other, had an annual household income less than $60 000, had some college‐level education or above, and were married (Table 1).

**Table 1:** Sample characteristics

	YBCS (n = 310)	Relatives (n = 431)	*P* value
Demographics			
Age (mean ± SD^a^)	51.4 ± 5.8	43.4 ± 11.9	<0.001
Race (N, %^b^)			
White/other	242 (78.1%)	344 (79.8%)	
Black	68 (21.9%)	87 (20.2%)	0.629
Education (N, %^b^)			
Elementary school only	0 (0%)	2 (0.5%)	
High school (grades 9‐11)	2 (0.7%)	9 (2.1%)	
Compulsory education (K‐12)	56 (18.3%)	61 (14.4%)	
Some college	113 (36.9%)	157 (37.0%)	
Completed college	74 (24.2%)	126 (29.7%)	
Postgraduate degree	61 (19.9%)	69 (16.3%)	0.109
Employment (N, %^b^)			
Full time	157 (50.7%)	237 (57%)	
Part time	42 (13.5%)	64 (15.4%)	
Unemployed/other^c^	111 (35.8%)	115 (27.6%)	0.357
Income (N, %^b^)			
<$20 000	24 (8.7%)	51 (13.2%)	
$20 000‐$39 999	42 (15.2%)	84 (21.8%)	
$40 000‐$59 999	50 (18.1%)	68 (17.6%)	
$60 000‐$79 999	51 (18.4%)	53 (13.7%)	
$80 000‐$99 999	30 (10.8%)	36 (9.3%)	
$100 000‐$119 999	29 (10.4%)	33 (8.5%)	
>$120 000	51 (18.4%)	61 (15.8%)	0.103
Marital status (N, %^b^)			
Married/life partner	212 (68.4%)	257 (59.8%)	
Single/divorced/widowed	98 (31.6%)	173 (40.2%)	0.020
Lives alone	44 (14.2%)	60 (13.9%)	0.998
Clinical characteristics			
Years since 1st diagnosis (mean ± SD^a^)	11.6 ± 4.0	N/A^d^	
Anxiety (N, %^b^)	89 (29.1%)	115 (27.1%)	0.604
Depression (N, %^b^)	84 (27.5%)	111 (26.1%)	0.731
Stressors			
Cost‐related lack of access to care (N, %^b^)	56 (18.1%)	82 (19.2%)	0.779
Fear of cancer recurrence (mean ± SD^a^)	3.4 ± 1.6	N/A^d^	
Perceived breast cancer risk (mean ± SD^a^)	N/A^d^	4.7 ± 2.0	
Resources			
Self‐efficacy (mean ± SD^a^)	5.9 ± 1.1	N/A^d^	

a Standard deviation.

b Valid percentages.

c Student, retired, housewife.

d N/A: not applicable.

Participants reported high levels of family support on the three family support scales (ie, communication, support in illness, and coherence) (observed range: 5.4‐6.1) and the family support index (YBCS: 5.6 ± 0.9; relatives: 5.7 ± 0.9) (Table 2). Relatives reported significantly higher family communication compared to YBCS. White/Other participants (YBCS and relatives combined) reported a higher score on the Family Support in Illness scale compared to Black participants (6.0 ± 1.1 vs. 5.8 ± 1.2, *P* = 0.001); this difference was not observed in the other two scales. Family support in illness was also higher in triads than dyads (Table 3).

**Table 2:** Family support in YBCS and relatives

	YBCS (N = 310) mean ± SD^a^	Relatives (N = 431) mean ± SD^a^	*P* value
Family communication	5.4 ± 1.1	5.6 ± 1.1	0.040
Family support in illness	6.0 ± 1.1	6.1 ± 1.0	0.087
Family coherence	5.4 ± 0.8	5.5 ± 0.8	0.688
Family Support Index	5.6 ± 0.9	5.7 ± 0.9	0.112

a Standard deviation.

**Table 3:** Mean family support in family units

	Dyads (n = 189) mean ± SD^a^	Triads (n = 121) mean ± SD^a^	*P* value
Family communication	5.4 ± 1.2	5.6 ± 1.0	0.437
Family support in illness	6.0 ± 1.1	6.2 ± 1.0	0.038
Family coherence	5.4 ± 0.8	5.5 ± 0.7	0.287
Family Support Index	5.6 ± 0.9	5.7 ± 0.8	0.134

a Standard deviation.

### 3.2 Predictors of family support in dyads and triads—APIM

#### 3.2.1 Dyadic findings

APIM with 189 dyads (YBCS and one relative) identified significant *actor effects* in YBCS and relatives, but no *partner effects* (Table 4). First, we tested an unconstrained dyadic model, which allowed *actor* and *partner effects* to differ between YBCS and relatives and found significant *actor effects*. As this dyadic model was a saturated model without fit indices, we tested an additional model where we constrained the *actor effects* to be equal to each other and the *partner effects* also to be equal to each other. No constraints were placed on dyad‐level predictors, that is, years since diagnosis, race, and degree type; on variables measured only in YBCS, that is, fear of recurrence and self‐efficacy. Chi‐square difference tests revealed that the effects did not differ across YBCS and relatives in the dyadic model (Χ^2^_diff_ = 15.390, *df* = 18, *P* = 0.6350). Thus, we report the constrained model results in Table 4. Similar to the unconstrained model, only *actor effects* were significant; a prior diagnosis of depression and cost‐related lack of access to care were associated with lower family support for YBCS and relatives. The strength of the *actor effects* between depression and cost‐related lack of access to care did not differ between YBCS and relatives. More years since diagnosis and higher self‐efficacy were associated with higher family support for YBCS, whereas Black race was associated with higher family support in relatives.

**Table 4:** Actor‐partner interdependence models^

Actor effects	Dyads		Triads	
	Estimate	*P* value	Estimate	*P* value
YBCS				
First‐degree relationship*	−0.166	0.364	0.191	0.226
Age	0.001	0.788	0.011	0.502
Black race*	0.285	0.068	0.014	0.949
Education	0.082	0.053	−0.073	0.329
Income	−0.010	0.308	0.008	0.573
Single relationship status	−0.159	0.175	−0.082	0.724
Lives alone	−0.103	0.491	0.101	0.714
Years since diagnosis*	**0.040**	**0.015**	**−0.050**	**0.031**
Anxiety	−0.198	0.075	−0.212	0.250
Depression	**−0.282**	**0.014**	−0.308	0.098
Cost‐related lack of access to care	**−0.302**	**0.007**	**−0.395**	**0.046**
Fear of recurrence*	0.048	0.286	0.025	0.649
Self‐efficacy*	**0.288**	**<0.001**	**0.303**	**<0.001**
Perceived risk	−0.004	0.857	−0.004	0.911
Relatives				
First‐degree relationship*	0.098	0.572	0.219	0.092
Age	0.001	0.788	0.001	0.697
Black race*	**0.419**	**0.005**	**0.424**	**0.022**
Education	0.082	0.053	0.057	0.171
Income	−0.010	0.308	0.002	0.807
Single relationship status	−0.159	0.175	−0.087	0.340
Lives alone	−0.103	0.491	0.113	0.408
Years since diagnosis*	−0.002	0.883	−0.023	0.224
Anxiety	−0.198	0.075	0.119	0.265
Depression	**−0.282**	**0.014**	−0.025	0.819
Cost‐related lack of access to care	**−0.302**	**0.007**	**−0.225**	**0.016**
Perceived risk	0.004	0.857	−0.026	0.198
Partner effects†				
YBCS → relative				
Age	0.007	0.202	**0.032**	**0.017**
Depression	−0.168	0.148	**−0.369**	**0.017**
Self‐efficacy*	0.067	0.309	**0.116**	**0.047**
Relative → YBCS				
Income	0.010	0.298	**0.019**	**0.034**

Unstandardized estimates (B) and standard errors (SE) reported.

^The dependent variable of the APIM analyses is the Family Support Index (PCA of the three scales); boldface indicates *P *<* *0.05.

†Only significant (*P *<* *0.05) partner effects in the triad data are reported along with the complementary nonsignificant results in the dyadic analysis; no significant (*P *<* *0.05) partner effects were observed in dyadic data.

*Dyad‐level predictors were degree type, race, and years since diagnosis; fear of recurrence and self‐efficacy were reported only by YBCS.

#### 3.2.2 Triadic findings

With the exception of the CFI (0.616), model fit indices were acceptable in the APIM triad analyses (nonsignificant chi‐square, *P* = 0.0552; X^2^/*df* ratio = 1.18; RMSEA = 0.038; SRMR = 0.057). APIM with 121 triads (YBCS and two relatives) identified significant *actor* and *partner effects* (Table 4). YBCS’ self‐efficacy was associated with higher YBCS family support. YBCS having cost‐related problems to accessing care and more years since diagnosis were associated with lower YBCS family support. Black race in relatives was associated with higher relative family support, while relatives’ cost‐related lack of access to care was associated with relatives’ lower family support. Four *partner effects* were identified in triads. YBCS’ prior diagnosis of depression was associated with relatives’ lower family support; YBCS’ older age and higher self‐efficacy were associated with relatives’ higher support; and relatives’ higher income was associated with YBCS’ higher support.

## 4 DISCUSSION

Early onset breast cancer can have a profound impact on cancer patients and their families. YBCS are a special group who have to manage both their own disease and their family roles. Their biological relatives also have to realize, accept, and manage a higher breast cancer risk. Family support is a valuable resource that can help address these challenges.

### 4.1 Family support in YBCS and relatives

Relatives reported higher family communication compared to YBCS, possibly due to YBCS’ unmet communication needs, especially for illness‐related issues.^50^, ^51^ White/Other participants (YBCS and relatives combined) reported higher family support at times of illness compared to Black participants. This could be partly because Black YBCS were significantly more likely to invite relatives living further than 50 miles away to participate in the study with them (data shown elsewhere),^25^ which could affect tangible support offered and received at times of illness. Triads reported higher family support at times of illness compared to dyads, presumably because it can be more difficult to find support from others at that time. Participating in the study with two vs one relative may indicate a stronger and larger support network.

### 4.2 Predictors and interdependence of family support

APIM examined both *actor* and *partner effects*, with the former being primary predictors of family support. *Partner effects* were observed only in triads, possibly due to greater chances of one person affecting the other person's responses.

#### 4.2.1 Self‐efficacy

Consistent with other studies, YBCS’ breast cancer self‐efficacy was an important predictor of their own family support (*actor effect*).^52^, ^53^ In dyads and triads, YBCS with more confidence in their ability to manage demands associated with breast cancer reported higher family support. Self‐efficacy is a key resource for cancer survivors associated with important outcomes, such as better mental health^54^ and higher quality of life.^55^ A novel finding of our study was that YBCS’ higher self‐efficacy had a significant *partner effect* on relatives’ perceived family support in triads. Relatives may find it easier to help YBCS who have higher self‐efficacy and fewer needs, as it may be less burdensome. YBCS who are better able to manage disease‐related stressors by themselves may feel more self‐reliant, creating less strains and demands on their family. YBCS with lower self‐efficacy is a group at risk for adverse outcomes and warrants further assessment and early intervention. In contrast, YBCS with higher self‐efficacy could be a resource for their relatives, who may also benefit from support in managing their own anxiety about cancer risk. This reciprocal relationship merits more investigation.

#### 4.2.2 Cost and access to care

About one in five YBCS and one in five relatives reported that there was a time during the past 12 months that they needed medical care but could not get it due to high out‐of‐pocket costs. Higher income is usually associated with availability of expendable resources to address illness‐related expenses.^7^, ^56^ Cancer may cause significant financial burdens and high out‐of‐pocket costs for YBCS; lack of support and available resources at times of need may further deter YBCS from accessing care. Relatives with higher income had a positive *partner effect* on YBCS’ family support in triads, possibly because they are considered an actual or potential resource to YBCS, accounting for YBCS perceiving higher support from them.

#### 4.2.3 Depression

YBCS with a prior diagnosis of depression reported lower family support (*actor effect*). Depression during diagnosis and treatment worsens for some breast cancer patients, especially for those lacking a partner or other forms of support.^57^, ^58^ Relatives who reported lower family support were also more likely to report a prior diagnosis of depression (*actor effect* in dyadic relationships) or to be associated with an YBCS with depression (*partner effect* in triadic relationships). The *partner effects* of YBCS’ prior diagnosis of depression indicate that YBCS’ depressive symptoms may influence relatives’ perceived family support. Relatives are expected to provide support to cancer survivors, although they also experience stressors and need support^59^; when relatives are depressed, they may perceive receiving less support, possibly as appreciation for their efforts.^60^ In a prior APIM analyses with a different sample of cancer survivors and their family caregivers, we also identified significant *longitudinal partner effects* between cancer patients’ depression and their family caregivers.^61^ Helping YBCS and relatives identify and manage depression is an important intervention area. However, we acknowledge that our findings may be influenced by participants’ recall bias, since we measured anxiety and depression with single‐item questions asking participants to recall what was told to them.

#### 4.2.4 Race

The activation of family support, which often increases with the burden of illness, can strengthen family relationships but can also strain network ties. In the face of a cancer diagnosis, family resources may be more readily available for some YBCS, or conversely not available for others, bringing up any differences in perceived family support that may have existed prediagnosis. Relatives of Black YBCS may hold strong beliefs about their familial obligations, due to strong familial and community orientations.^62^ Expressions of familialism and collectivism are more evident in Blacks than other racial groups, due to a traditional caregiving ideology, related to collectivism in social relationships.^63^ However, this finding should be interpreted with caution, since there were a smaller number of Black relatives in the study and the other group included White YBCS and a small proportion of YBCS of other ethnic/racial background.

#### 4.2.5 Length of survivorship

Finally, being a longer‐term cancer survivor was associated with less family support reported by YBCS in triadic relationships. Since YBCS in the study were diagnosed on average 11 years prior, family members may assume that YBCS need less support over time. This may not necessarily be accurate, as some YBCS may have to cope with late effects of cancer treatment or pervasive fear of cancer recurrence.^64^, ^65^ YBCS’ age had a positive *partner effect* on relatives’ family support, presumably because some longer‐term cancer survivors focus less on their own needs and more on the needs of their families.^66^

### 4.3 Strengths and limitations

Limitations include self‐reporting and recruitment preferences. Relatives were invited directly from YBCS, which assumes that relatives in good relationship with the YBCS were prioritized; this may explain the small range of scores in the family support index (5.3‐6.1; possible range 1‐7). The study invited up to two relatives per YBCS for comparable family units, although some YBCS may be receiving support from larger networks. Stressors, that is anxiety and depression, were assessed with single‐item measures instead of lengthier instruments to reduce overall burden, but responses may be influenced by recall bias. YBCS of “other” racial/ethnic backgrounds were combined with White YBCS, thus findings related to race may not be generalized. Finally, we did not assess whether YBCS were receiving treatment, which may have implications for family support they needed at the time of the survey.

Future research with YBCS and relative triads may consider a conceptually meaningful way to distinguish relatives as part of the study design and in line with the study aims. For example, the study could have required YBCS to designate one relative who provides more support. However, prior work of the research team demonstrated that cancer patients have difficulty and may feel uncomfortable about making a choice and indicating who provides more support. Thus, we consider that extending the term “family” to include more than two people and attending to the challenges associated with these complex analyses are significant strengths of our study.

We examined self‐efficacy as a predictor of family support, which is not the way it has been traditionally examined in prior literature. Our findings indicate a strong correlation between family support and patients’ self‐efficacy (ie, patients’ confidence in their own ability to manage the disease). Traditionally, family support has been examined as a predictor of self‐efficacy. An alternative hypothesis suggests that the ability of people to provide support is associated with the characteristics of the person needing support. It may be more difficult to support cancer patients who have low self‐efficacy (ie, low self‐confidence) in their ability to manage the disease. It is possible that the support person may need to spend more time bolstering or encouraging the patient with low self‐efficacy, which may become burdensome over time. Further research is needed in this area. Due to the cross‐sectional nature of the data, we can only report the significant correlation between self‐efficacy and support rather than confirm causation from one variable to the other variable.

### 4.4 Implications

Family support enhances the long‐term physical and mental well‐being of cancer patients and their family members.^6^, ^16^, ^67^, ^68^, ^69^ It also provides greater cohesion and strengthens the interpersonal contacts among breast cancer patients and their relatives.^67^ Our findings demonstrate the interdependence of family support between YBCS and their close biological relatives. APIM provided valuable insights into complex family relationships taking also into account relatives’ increased breast cancer risk due to possible hereditary susceptibility. Consistent with our theoretical framework, self‐efficacy and access to care were important resources that influence family support. Perceived stressors associated with early onset breast cancer and availability of resources may affect the level of support family members are willing to give and receive to each other. Due to the interdependence among YBCS and their relatives, supportive programs need to focus on the YBCS‐relative as the unit of care and include family‐based interventions that enhance each person's self‐efficacy and access to high‐quality services.^70^, ^71^, ^72^ Existing evidence‐based interventions for cancer patients and their family caregivers^72^, ^73^, ^74^, ^75^ could be adapted to address the needs of YBCS and their relatives. It is also important for researchers and clinicians to work together and develop technology‐based dyadic interventions that are cost‐effective and accessible to a large number of families.^76^, ^77^, ^78^

## CONFLICT OF INTEREST

None declared.
